# On the Microcrystal Structure of Sputtered Cu Films Deposited on Si(100) Surfaces: Experiment and Integrated Multiscale Simulation

**DOI:** 10.3390/molecules28124786

**Published:** 2023-06-15

**Authors:** Guo Zhu, Mengxin Han, Baijun Xiao, Zhiyin Gan

**Affiliations:** 1 School of Mechanical & Electrical Engineering, Hunan City University, Yiyang 413000, China; 15176105972@163.com (M.H.); xbjcai@sina.cn (B.X.); 2 School of Mechanical Science & Engineering, Huazhong University of Science & Technology, Wuhan 430074, China; zhiyingan@126.com

**Keywords:** magnetron sputtering deposition, application-oriented simulation, sputtered particle transport, Cu/Si film deposition, microcrystal structure

## Abstract

Sputtered Cu/Si thin films were experimentally prepared at different sputtering pressures and characterized using X-ray diffraction (XRD) and an atomic force microscope (AFM). Simultaneously, an application-oriented simulation approach for magnetron sputtering deposition was proposed in this work. In this integrated multiscale simulation, the sputtered atom transport was modeled using the Monte Carlo (MC) and molecular dynamics (MD) coupling method, and the deposition of sputtered atoms was simulated using the MD method. This application-oriented simulation approach was used to simulate the growth of Cu/Si(100) thin films at different sputtering pressures. The experimental results unveiled that, as the sputtering pressure decreased from 2 to 0.15 Pa, the surface roughness of Cu thin films gradually decreased; (111)-oriented grains were dominant in Cu thin films and the crystal quality of the Cu thin film was gradually improved. The simulation results were consistent with the experimental characterization results. The simulation results revealed that the transformation of the film growth mode from the Volmer–Weber growth mode to the two-dimensional layered growth mode resulted in a decrease in the surface roughness of Cu thin films; the increase in the amorphous compound CuSi_x_ and the hcp copper silicide with the decrease in the sputtering pressure was responsible for the improvement of the crystal quality of the Cu thin film. This work proposed a more realistic, integrated simulation scheme for magnetron sputtering deposition, providing theoretical guidance for the efficient preparation of high-quality sputtered films.

## 1. Introduction

Magnetron sputtering technology has been generally employed to prepare semiconductor, metal, magnetic, and optical films [1,2,3,4] due to its low deposition temperature, high film/substrate adhesion, and environmental friendliness. Recently, thin-film bulk acoustic resonators (FBARs), smart sensors and highly integrated metal-oxide-semiconductor field-effect transistors (MOSFETs) have received considerable attention for their potential application prospects in the fields of communication, smart homes, and digital technology. Magnetron sputtering technology has been widely used to prepare AlN films [5] in FBAR, environmentally sensitive films [6,7] in smart sensors, and interelectrode contact films [8,9] in MOSFET. The increasingly widespread application of magnetron sputtering technology in the micro-nano manufacturing field requires more accurate process control during the preparation of sputtered films. Accordingly, exploring the influence mechanism of process parameters on the properties of the sputtered film has great significance for the efficient fabrication of high-quality sputtered films.

It is known that magnetron sputtering deposition involves the transport processes of sputtered particles in the gas phase [10] and deposition processes of sputtered particles on the substrate [11]. Tuning sputtering process parameters results in the variation in transport processes of sputtered particles, leading to the alteration of incident energy and angle distributions of sputtered particles [12] when they reach the substrate surface and thus the microstructural changes of the sputtered film. Therefore, a comprehensive study on the transport and deposition processes of sputtered particles is important to explore the influence mechanism of process parameters on the properties of the sputtered film.

So far, the transport and deposition processes involved in magnetron sputtering deposition cannot be real-time monitored due to the limitation of experimental conditions. Consequently, repeated sample preparation and posteriori characterization is the main approach to exploring the relationship between process parameters and properties of the sputtered film [13]. On the other hand, numerical simulation can be used to compensate for the limitation of experimental conditions and reveal the relevant mechanism. Chu et al. [14] conducted MD simulation to investigate the effects of various process parameters on the deposition of Cu/Cu thin film using sputtering deposition. Taguchi et al. [15] emulated the growth of amorphous SiO_2_ thin film in reactive sputtering using MD simulation. In the MD models mentioned above, the initial incident energy and angle of film atoms were kept constant in a simulation cycle. Georgieva et al. [16] simulated the deposition of alloy oxide films in reactive sputtering using MD simulation. In this improved MD model, the initial energy of film atoms was assumed to obey Maxwellian distributions. Brault et al. [17,18] performed MD simulation to model the deposition of sputtered metal and nanocatalyst films using magnetron sputtering. In their simulations, an analytical model of the particle slowing down along straight-line trajectories [19] was adopted to calculate the incident energy of film atoms closer to the actual conditions. Given the oversimplification of the initial energy and angle of the film atoms in the above MD simulation, it is necessary to explore a more realistic simulation scheme for the growth of sputtered films.

In this work, sputtered Cu/Si thin films were experimentally prepared at different sputtering pressures and characterized using X-ray diffraction (XRD) and an atomic force microscope (AFM). Simultaneously, the transport processes of sputtered Cu atoms in the argon gas and the deposition processes of sputtered Cu atoms on the Si(100) substrate under different sputtering pressures were successively simulated using MC–MD coupling simulation [20] and MD simulation. The evolution mechanism of the microcrystal structure of the sputtered Cu film with the sputtering pressure was investigated based on the simulation results of this integrated multiscale simulation of combined transport and deposition processes. This work attempted to exploit a realistic simulation approach to more precisely characterize the physical processes involved in magnetron sputtering deposition, effectively improving the process development efficiency of high-quality sputtered films.

## 2. Results

### 2.1. Experimental Characterization Results

**Figure 1:**
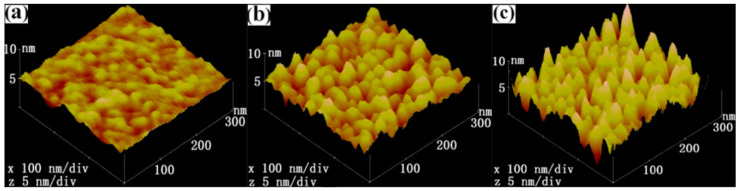
AFM morphology of Cu thin films prepared in the experiment at (**a**) 0.15 Pa, (**b**) 0.5 Pa, and (**c**) 2 Pa.

Figure 1 displays the surface topography of the sputtered Cu films at different sputtering pressures. It can be seen that, as the sputtering pressure decreased from 2 to 0.15 Pa, the surface roughness of the sputtered film gradually decreased, which agrees well with the experimental observation [21,22]. Thornton [23] proposed a structure zone diagram for magnetron sputtering deposition in which the structure of the sputtered film was determined by the sputtering pressure and temperature. Indeed, the effects of the sputtering pressure on the film structure are attributed to the variation in the energy deposited on the growing film surface [24]. Accordingly, Anders [25] deemed that the structure of the sputtered film was governed by the thermal effects and kinetic effects, proposing a modified SZD. According to the SZD models proposed by Thornton and Anders, at Zone 1 (T_h_/T_m_ < 0.3, where T_h_ and T_m_ are the film growth temperature and melting point of film materials, respectively), the surface roughness of the sputtered films gradually decreased with the decrease in the sputtering pressure (generalized energy) when the temperature was kept constant.

**Figure 2:**
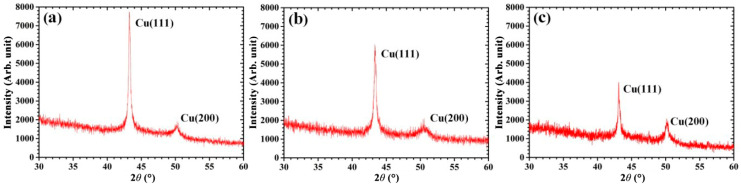
XRD patterns of Cu thin films prepared in the experiment at (**a**) 0.15 Pa, (**b**) 0.5 Pa, and (**c**) 2 Pa.

Figure 2 shows the XRD patterns of the sputtered Cu thin films prepared at 0.15, 0.5, and 2 Pa. As shown in Figure 2, the (111) preferred orientation can be observed in the sputtered Cu films prepared under all three sputtering pressures. Indeed, for the case of the Cu/Si film deposition, (111)-oriented grains preferentially grew on the clean Si(100) surface [26], while (100)-oriented grains preferentially grew on the hydrogenated Si(100) surface [27,28,29]. At 0.15 Pa, the diffraction intensity of the (111) plane was significantly higher than that of the (100) plane. As the sputtering pressure gradually increased from 0.15 to 2 Pa, the diffraction intensity of the (111) plane gradually decreased, while the diffraction intensity of the (100) plane gradually increased. This indicates that the decrease in the sputtering pressure results in the increase in I<111>/I<100>, which denotes the ratio of the (111) orientation to the (100) orientation in the sputtered Cu film. Lim et al. [30] reached the same conclusion when they studied the variation in the crystalline structure of sputtered Cu thin films deposited at 6, 9, and 12 Pa, respectively. In addition, since the mean time to failure (TMF) of Cu interconnects in Si integrated circuits increased with I_<111>_/I_<100>_ [31], the decrease in the sputtering pressure might be beneficial for the increase in the TMF of Cu interconnects in Si integrated circuits.

Since the sputtered Cu film is not a monocrystal structure, the diffraction peaks in the XRD pattern have a specific width. The full width at half maxima (FWHM) of the diffraction peak is an important parameter to characterize the crystal quality of Cu thin films. The average grain size of the Cu thin film can be calculated using Scherrer’s formula [32]:

(1) $$D=\frac{K\lambda }{\beta \cos\theta }$$

where *D* is the average grain size in the direction perpendicular to the crystal plane (Å), *K* is the average crystallite shape factor, *λ* is the wavelength of the X-ray (Å), *β* denotes the width at half maximum (FHWM) of the respective XRD peak, and *θ* is the diffraction peak angle.

According to Scherrer’s formula, the grain size is inversely proportional to *β* (FHWM). As the sputtering pressure increased from 0.15 to 2 Pa, the FHWM of the (111) orientation in the Cu thin films increased from 0.38 to 0.59, while the FHWM of the (100) orientation in the Cu thin films decreased from 1.64 to 0.82. It can be found that the FHWM of the (111) orientation in Cu thin films deposited under three sputtering pressures was significantly smaller than that of the (100) orientation. This suggests that the average size of (111)-oriented grains in sputtering Cu thin films is greater than that of the (100)-oriented grains. Moreover, as the sputtering pressure increased from 0.15 to 2 Pa, the average size of (111)-oriented grains in the Cu thin films gradually decreased.

### 2.2. Simulation Results

**Figure 3:**
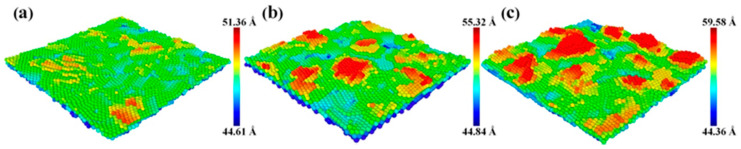
Surface morphology of Cu thin films deposited at (**a**) 0.15 Pa, (**b**) 0.5 Pa, and (**c**) 2 Pa [33].

Figure 3 depicts the surface morphology of Cu thin films deposited at 0.15, 0.5, and 2 Pa. From Figure 3, it can be seen that, as the sputtering pressure decreased from 2 to 0.15 Pa, the growth mode of the Cu thin film was gradually transformed from the Volmer–Weber growth mode to the two-dimensional layered growth mode, leading to the decrease in the surface roughness of the Cu thin film. By comparing Figure 1a and Figure 3a, Figure 1b and Figure 3b, and Figure 1c and Figure 3c, it can be found that, under the same sputtering pressure, the surface morphology of the Cu film obtained using molecular dynamics simulation was not exactly the same as that of the Cu film prepared by experiment. This is mainly due to the fact that MD simulation results actually display the surface morphology of the Cu film at the initial stage of the film deposition, and the area of the MD simulation domain (11.95 nm × 11.95 nm) is too small compared with that characterized by AFM (300 nm × 300 nm). The MD simulation results indicate that the surface roughness of the sputtered Cu film increased with the sputtering pressure, which is consistent with our experimental results.

**Figure 4:**
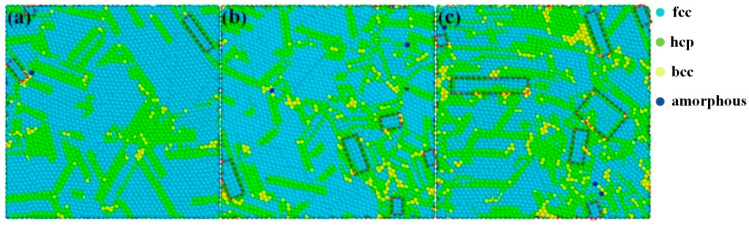
Microcrystal structure of Cu films deposited at (**a**) 0.15 Pa, (**b**) 0.5 Pa, and (**c**) 2 Pa. In each sub-graph, fcc, bcc, hcp, and amorphous structures are colored cyan, yellow, green, and blue, respectively, and the (100)-oriented grains are marked by brown rectangular frames.

Figure 4 shows the microcrystal structure at the particular cross-section (Z = 48 Å) of Cu thin films deposited on the Si(100) substrate at 0.15, 0.5, and 2 Pa, respectively. OVITO [34] was used to classify fcc, bcc, hcp, and amorphous structures, which are colored cyan, yellow, green, and blue, respectively. In each sub-graph of Figure 4, the (100)-oriented grains are marked by brown rectangular frames, and the remaining grains colored cyan are (111)-oriented. It can be found that (100)- and (111)-oriented grains are formed in Cu thin films deposited under all three sputtering pressures, and (111)-oriented grains are dominant in Cu thin films. With the decrease in the sputtering pressure, the proportion of (100)-oriented grains in Cu thin films decreases, while the proportion and size of (111)-oriented grains in Cu thin films both increase. Accordingly, our simulation results are consistent with the experimental results.

From the point of view of surface energy minimization, the deposited atoms arrange themselves preferentially in the most densely packed crystallographic plane to lower the total surface energy [35]. For the fcc copper, it happens to be the (111) plane. Moreover, the incident energy of Cu atoms ranges from a few tenths of eV to dozens of eV, while the surface binding energy and displacement threshold energy of Cu material are 3.49 eV [19] and 18 eV [36], respectively. Accordingly, some incident Cu atoms might induce near-surface cascade collisions [37], which might result in the variation in the growing surface of the sputtered film. Close-packed two-dimensional adatom (the sputtered atom deposited on the film surface) shells, such as heptamer, decamer, and dodecamer, are particularly stable due to the maximum number of bonds per atom [38]. These adatom shells are prone to survive under the bombardment of high-energy Cu atoms and ultimately coalesce to form (111)-oriented grains. Furthermore, as the sputtering pressure decreases, the increased incident energy enables deposited adatoms to migrate a longer distance on the growing film surface and occupy the most energetically favorable lattice sites. More violent bombardments of incident Cu atoms on the growing surface of the sputtered film reduce the formation probability of (100)-oriented grains. Consequently, the proportion and size of (111)-oriented grains in Cu thin films both increase with the sputtering pressure. With the decrease in the sputtering pressure, close-packed two-dimensional adatom nuclei can coalesce more easily on the flatter surface of the Cu thin film, forming larger (111)-oriented grains.

**Figure 5:**
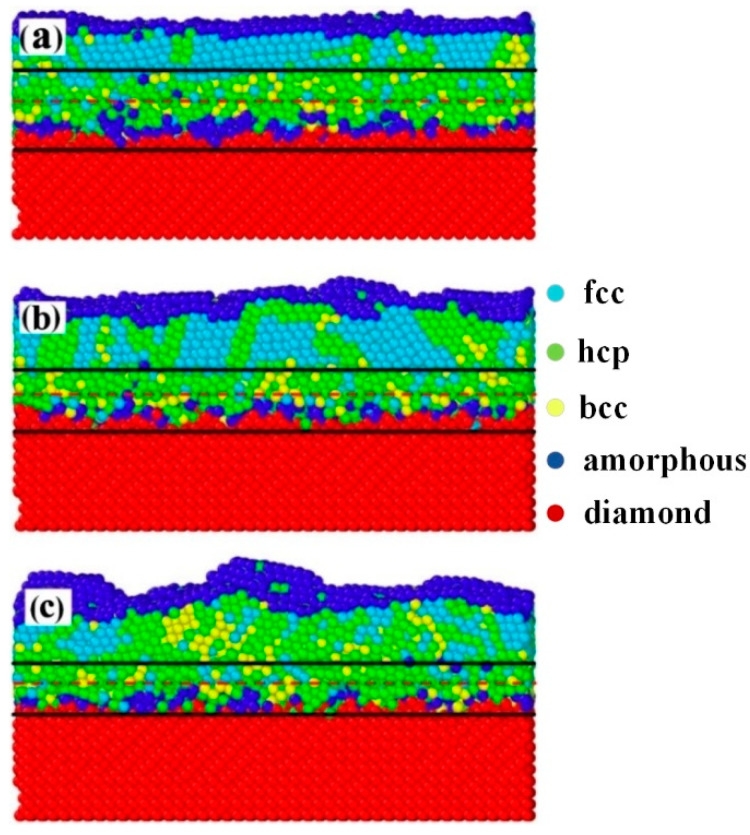
Microcrystal structure at the cross-section perpendicular to the surface of Cu films deposited at (**a**) 0.15 Pa, (**b**) 0.5 Pa, and (**c**) 2 Pa. In each sub-graph, fcc, hcp, bcc, amorphous, and diamond structures are colored cyan, green, yellow, blue, and red, respectively.

Figure 5 further shows the microcrystal structure at the cross-section perpendicular to the surface of Cu films deposited at 0.15, 0.5, and 2 Pa, respectively. In Figure 5, fcc, hcp, bcc, amorphous, and diamond structures are colored cyan, green, yellow, blue, and red, respectively. In each sub-graph, the Cu/Si interface width is labeled with two black lines, and the initial substrate surface is marked by a red dashed line. As shown in Figure 5, copper silicide with hcp and bcc structures can be found in the Cu/Si interface, and the hcp copper silicide is dominant in the Cu thin films deposited at all three sputtering pressures. The copper silicide with hcp and bcc structures had also been observed in experiments [39,40]. The proportion of the hcp copper silicide (ζ-phase) gradually increased as the sputtering pressure decreased from 2 to 0.15 Pa. Indeed, the lattice parameters of the ζ-phase copper silicide (CuSi_12_) and Cu (111) were 2.5599 [39] and 2.5558 Å, respectively. Accordingly, the lattice mismatch between the ζ-phase(0002) and Cu (111) planes was less than 0.1%. Accordingly, the increase in the hcp copper silicide in the Cu/Si interface might be responsible for the increase in the proportion and size of (111)-oriented grains in sputtered Cu thin films.

**Figure 6:**
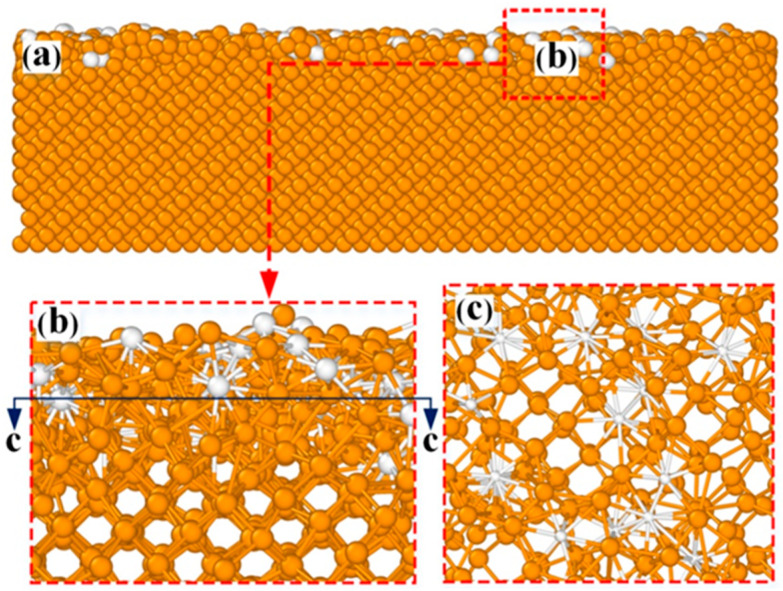
Microstructure of Cu/Si interface after 500 Cu atoms are deposited onto the Si(100) surface at 0.15 Pa. (**a**) Whole structure of the Cu/Si system; (**b**) Local enlarged view of the Cu/Si interfacial region marked by the red rectangle frame in sub-graph (**a**); (**c**) Microstructure of Cu/Si interface viewed along the c-c direction in sub-graph (**b**). In each sub-graph, Cu and Si atoms are colored white and orange, respectively.

More importantly, copper silicide with an amorphous structure arose at the bottom of the Cu/Si interface, which suggests that this amorphous structure is formed at the initial stage of Cu film growth. To explore the component of the amorphous phase, the microstructure of the Cu/Si interface at the initial stage of Cu film growth was further analyzed in detail. Figure 6 depicts the microstructure of the Cu/Si interface after 500 Cu atoms were deposited onto the Si(100) surface at 0.15 Pa. In Figure 6, Si and Cu atoms are colored orange and white, respectively. As shown in Figure 6b, the near-surface lattice sites of the Si substrate have been disordered due to the implantation of Cu atoms. Figure 6c further unveils the formation of the amorphous compound CuSi_x_. This amorphous compound had also been observed at the phase boundary towards the Si-rich side in the experiment [41,42], which is probably a variant of the *η*″-phase [41] and serves as a buffer layer to reduce the internal stress caused by the bond mismatch between the Cu/Si interface and the substrate [42]. As shown in Figure 6, the amorphous structure and the hcp copper silicide both increased as the sputtering pressure decreased from 2 to 0.15 Pa, which suggests that the existence of the CuSi_x_ compound promotes the formation of hcp copper silicide and thus the enlargement of (111)-oriented grains of the Cu thin film.

## 3. Materials and Methods

### 3.1. Experimental Method

Cu thin films were fabricated on Si(100) substrates using a DC magnetron sputtering system with a nonparallel, off-axis target-substrate configuration. A copper target was used as the sputtering source, and its purity and diameter were 99.995% and 50.8 mm, respectively. The intrinsic monocrystal (100) silicon wafers were utilized as substrates; their diameter was 50.8 mm. The copper target and silicon wafers were produced by the YPCCTECK corporation (Haidian, BJ, China). Argon gas was adopted to be the sputtering gas. Before the sputtering deposition, the silicon wafer was successively washed in acetone and anhydrous ethanol for 5 min using an ultrasonic cleaner to remove grease from the substrate surface. After that, the silicon wafer was soaked in 5% HF solution for 2 min to remove the oxide layer on the substrate surface and then dipped into anhydrous ethanol and ultrasonically cleaned for 5 min. The silicon wafer was then fixed onto the substrate holder of the magnetron sputtering system. The base pressure of the sputtering chamber was pumped down to 5 × 10^−4^ Pa using a molecular pump. Three experiments were performed in which the argon gas pressure was maintained at 0.15, 0.5, and 2 Pa, and the target voltage was kept at 378, 344, and 338 V, respectively. The film deposition time was set to 10 min. The microcrystal structure of sputtered Cu thin films was characterized using a SmartLab-SE X-ray diffractometer (XRD) produced by the Rigaku Corporation (Akishima, TKY, Japen). The surface morphology of the sputtered Cu films was characterized using a contact atomic force microscope (AFM) produced by the Veeco corporation (Plainview, NY, USA).

### 3.2. MC–MD Simulation of the Transport of Sputtered Cu Atoms

The transport processes of sputtered Cu atoms in the argon gas were simulated using the MC–MD coupling method. In our simulation, the temperature of the sputtering gas was set to 300 K.

**Figure 7:**
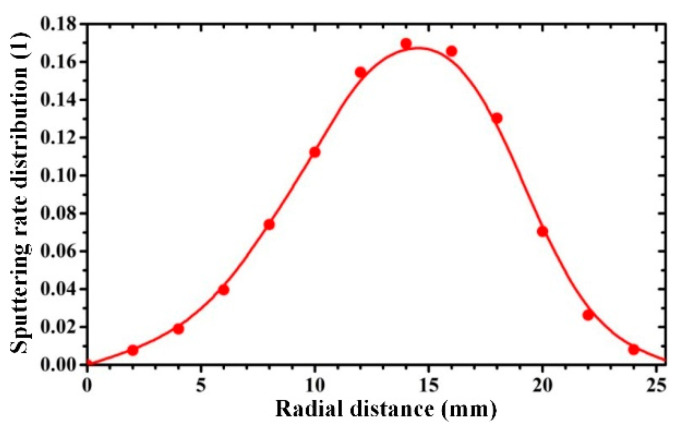
Radial sputtering possibility distribution of the copper material.

The sputtering possibility of the target material is dependent on the magnetic field intensities on the target surface and can be given by [43]

(2) $$P(r)\propto \frac{B_{r}(r)}{B_{r}^{2}\left(r\right)+B_{z}^{2}\left(r\right)}$$

where *r* is the radial distance between the initial position of the sputtered atom and the target center; *B_r_* and *B_z_* denote the horizontal and vertical magnetic field intensities, respectively. A Hall sensor was utilized to measure the magnetic field intensity (*B*_r_ and *B*_z_) along the radial direction of the target surface [44], and its margin of error and maximal measuring range were 2% and 2400 mT, respectively. The radial sputtering rate distribution of the copper material is displayed in Figure 7.

The sputtering probability of an atom located at a distance of *r* from the target center can be calculated through Equation (2). Accordingly, the emission position coordinates of the sputtered atom were generated by a random number *n* distributed uniformly between 0 and 1.

(3) $$x_{0}=r\cos(2\pi n)$$

(4) $$y_{0}=r\sin(2\pi n)$$

The initial energy and polar emission angle of the Cu atom ejected from the target surface were chosen using the rejection technique from the Thompson distribution [45] and Yamamura’s angular distribution [46], respectively. The azimuth emission angles of sputtered atoms (*φ*) were sampled uniformly between 0 and 2*π* due to their symmetry.

**Figure 8:**
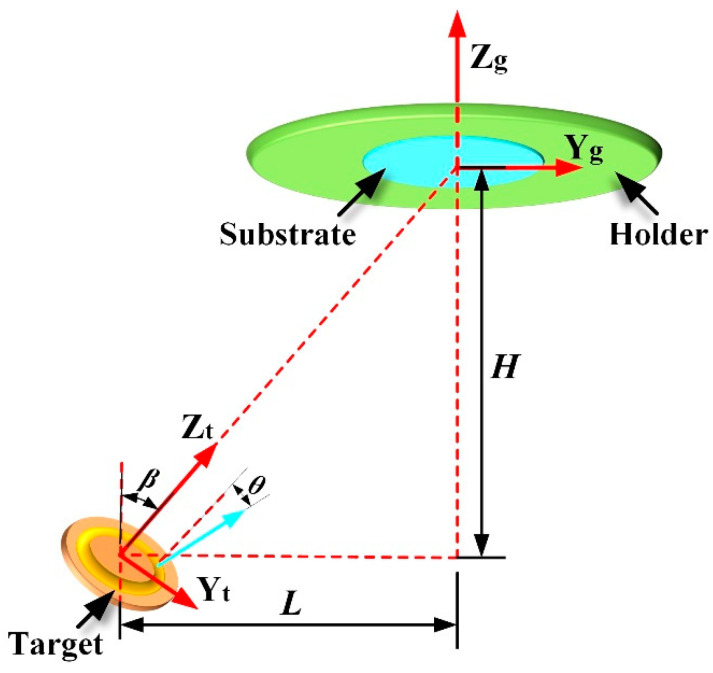
Geometric structure of the magnetron sputtering system with nonparallel off-axis target-substrate configuration.

Figure 8 shows the target-substrate configuration of the magnetron sputtering system. In Figure 8, *β* denotes the including angle between the target surface normal and the substrate normal; *H* and *L* represent the vertical and horizontal distances between the target center and the substrate center. Herein, *β*, *H*, and *L* are set to 25°, 60 mm, and 112 mm, respectively. In Figure 8, a local coordinate system X_t_Y_t_Z_t_ and a global coordinate system X_g_Y_g_Z_g_ were introduced to facilitate the analysis. The origins of X_t_Y_t_Z_t_ and X_g_Y_g_Z_g_ were located at the target center and the substrate center, respectively; the Z_t_ axis and Z_g_ axis were along the target surface normal and the substrate surface normal, respectively. In the local coordinate system X_t_Y_t_Z_t_, the initial position of a sputtered atom was labeled as point *m* (*x*_0_, *y*_0_, *z*_0_), while the initial velocity of the sputtered atom was marked by the vector *V* (*v*_x0_, *v*_y0_, *v*_z0_). Based on the geometric relationship between X_t_Y_t_Z_t_ and X_g_Y_g_Z_g_, the initial position and velocity of the sputtered atom calibrated in X_g_Y_g_Z_g_ can be given by

(5) $$\left[\begin{array}{c} x_{1}\\ y_{1}\\ z_{1} \end{array}\right]=\left[\begin{array}{ccc} 1 & 0 & 0\\ 0 & \cos\beta & \sin\beta \\ 0 & -\sin\beta & \cos\beta \end{array}\right]\left[\begin{array}{c} x_{0}\\ y_{0}\\ z_{0}=0 \end{array}\right]+\left[\begin{array}{c} 0\\ -L\\ -H \end{array}\right]$$

(6) $$\left[\begin{array}{c} v_{x1}\\ v_{y1}\\ v_{z1} \end{array}\right]=\left[\begin{array}{ccc} 1 & 0 & 0\\ 0 & \cos\beta & \sin\beta \\ 0 & -\sin\beta & \cos\beta \end{array}\right]\left[\begin{array}{c} v_{x0}\\ v_{y0}\\ v_{z0} \end{array}\right]=\left[\begin{array}{ccc} 1 & 0 & 0\\ 0 & \cos\beta & \sin\beta \\ 0 & -\sin\beta & \cos\beta \end{array}\right]\left[\begin{array}{c} V\sin\theta \cos\phi \\ V\sin\theta \sin\phi \\ V\cos\theta \end{array}\right]$$

When the initial status data of sputtered Cu atoms were obtained, the MC–MD coupling method was adopted to simulate their transport processes in the argon gas and to further calculate the incident energy and polar angle distributions of the Cu atoms deposited on the Si substrate. The specific procedure of the MC–MD coupling method was described in our previous paper [20].

### 3.3. MD Simulation of the Growth of Sputtered Cu Film on Si Substrate

The deposition of sputtered Cu atoms onto the Si(100) surface was modeled using the molecular dynamics code LAMMPS [47]. The interaction potential functions between atoms are listed in Table 1. The dimensions of the simulation domain were set to 22a × 22a × 16a (a denotes the lattice constant of Si). The simulation domain was composed of a substrate and a vacuum space. The sizes of the substrate and the vacuum space were set to 22a × 22a × 6a and 22a × 22a × 10a, respectively. The *z*-axis was perpendicular to the Si(100) surface. A non-periodic and fixed boundary condition was applied in the Z direction, and periodic boundary conditions were used in the X and Y directions. The bottommost four layers of substrate atoms were defined as fixed atoms. Si atoms located at 5 to 12 layers of substrate were classified as thermostat atoms. The velocities of thermostat atoms were rescaled every 10 fs to keep the temperature of the thermostat atoms at 300 K. The topmost 12 layers of substrate atoms were Newtonian atoms. The standard velocity Verlet algorithm was employed to solve the velocities of the Newtonian atoms, and the time-step was set to 1 fs [48].

In each simulation, 25,000 Cu atoms were released successively every 2000 time-steps [26]. Each Cu atom was initially placed one lattice distance (5.43 Å) above the film surface. The initial XY coordinates of these Cu atoms were chosen randomly from the region of 22a × 22a. The incident energy and polar angle of each Cu atom were selected base on the incident energy and polar angle distributions calculated using the MC–MD coupling simulation. The incident azimuth angles of these Cu atoms were distributed uniformly between 0 and 2π. Once the deposition processes of 25,000 Cu atoms were accomplished, a relaxation of 1000 ps enabled the deposited Cu film to reach thermal equilibrium.

## 4. Conclusions

In this work, sputtered Cu/Si(100) thin films were experimentally prepared at different sputtering pressures and characterized using X-ray diffraction (XRD) and an atomic force microscope (AFM). Simultaneously, an integrated simulation of combined transport and deposition processes was performed to investigate the deposition of sputtered Cu/Si(100) thin films using magnetron sputtering at 0.15, 0.5, and 2 Pa. Experimental characterization results indicated that, for all three Cu thin film samples, the diffraction intensity of the (111) plane was significantly higher than that of the (100) plane, while the FHWM of the (111) orientation was obviously smaller than that of the (100) orientation. As the sputtering pressure decreased from 2 to 0.15 Pa, the FHWM of the (111) orientation gradually decreased. Simulation results manifested that (111)-oriented and (100)-oriented grains were formed in Cu thin films deposited at all three sputtering pressures, and (111)-oriented grains were dominant. The proportion and size of (111)-oriented grains in Cu thin films both increased with the decrease in sputtering pressure from 2 to 0.15 Pa. According to the analysis based on Scherrer’s formula, the simulation results agreed with the experimental characterization results. The surface morphology of Cu thin films and the microstructure of the Cu/Si interface were further analyzed based on simulation results. As the sputtering pressure decreased from 2 to 0.15 Pa, the growth mode of the Cu thin film was gradually transformed from the Volmer–Weber growth mode to the two-dimensional layered growth mode, and the decrease in the surface roughness might be beneficial to the enlargement of (111)-oriented grains. At all three sputtering pressures, the Cu/Si interface consisted of copper silicide with hcp and bcc structures, and hcp copper silicide was dominant in the Cu/Si interface. Furthermore, the amorphous compound CuSi_x_ was found between the Cu/Si interface and the Si substrate. More importantly, (111)-oriented grains of Cu thin films, the hcp copper silicide, and the amorphous compound CuSi_x_ all increased with the decrease in the sputtering pressure. This suggested that the stacking structure of hcp copper silicide/CuSi_x_/Si was in favor of the growth of (111)-oriented grains in the Cu thin film. The underlying mechanism was interpreted with the point of view of lattice matching and internal stress relief. This work is expected to propose a more realistic, integrated simulation of combined transport and deposition processes for magnetron sputtering deposition, providing theoretical reference for the efficient preparation of high-quality sputtered films.

## Figures and Tables

**Table 1:** Interaction potential functions between atoms.

Interaction	Si–Si	Cu–Cu	Cu–Si
Potential	Tersoff [49]	EAM [50]	Extended Tersoff [51,52]

## Data Availability

Not applicable.
